# Early high-frequency spinal cord stimulation modulates the ROS/p38 MAPK/NF-κB and CXCL10/CXCR3 pathways to alleviate neuropathic pain and promote spinal cord injury repair

**DOI:** 10.3892/ijmm.2026.5956

**Published:** 2026-08-13

**Authors:** Sitong Su, Tao Liu, Honghui Lei, Yang Yu, Caizhi Ma, Xinglang Wang, Haoyuan Chen, Zebin Huang, Meiling Cheng, Tongtong Qian, Fangyong Wang

**Affiliations:** 1 School of Rehabilitation, Capital Medical University, Beijing 100069, P.R. China; 2 Department of Spine Surgery, Beijing Bo'ai Hospital, China Rehabilitation Research Center, Capital Medical University, Beijing 100068, P.R. China; 3 Department of Rehabilitation Engineering, China Rehabilitation Science Institute, Beijing 100068, P.R. China; 4 School of Biological Science and Medical Engineering, Beihang University, Beijing 100191, P.R. China; 5 Rehabilitation Medicine Center, The Second Affiliated Hospital and Yuying Children's Hospital, Wenzhou Medical University, Wenzhou, Zhejiang 325000, P.R. China; 6 School of Rehabilitation Sciences and Engineering, University of Health and Rehabilitation Sciences, Qingdao, Shandong 266113, P.R. China; 7 Department of Orthopedics, Qilu Hospital, Cheeloo College of Medicine, Shandong University, Jinan, Shandong 250012, P.R. China; 8 School of Rehabilitation Medicine, Shandong University of Traditional Chinese Medicine, Jinan, Shandong 250355, P.R. China

**Keywords:** microglia, neuroinflammation, central sensitization, C-X-C motif chemokine ligand 10/C-X-C motif chemokine receptor 3, reactive oxygen species/p38 MAPK/NF-κB, neuropathic pain, spinal cord injury, high-frequency spinal cord stimulation

## Abstract

High-frequency spinal cord stimulation (HF-SCS) is an effective method for treating neuropathic pain (NP), but its specific mechanism of action in treating spinal cord injury (SCI) remains unclear. The present study aimed to explore the therapeutic effect of early HF-SCS in a rat model of SCI and its potential molecular mechanism. A Sprague-Dawley rat model of T10 spinal cord contusion was established and stimulation electrodes were implanted epidurally, which was followed by HF-SCS treatment (40% movement threshold at a frequency of 10 kHz). Through behavioral assessments, histopathological analysis and immunofluorescence staining, the present study demonstrated that HF-SCS markedly alleviated post-SCI NP, facilitated functional recovery and accelerated axonal regeneration and myelin repair. Mechanistic studies employing RNA sequencing, western blotting and immunofluorescence further revealed that HF-SCS exerted its neuroprotective effect by downregulating the reactive oxygen species/p38 MAPK/NF-κB signaling pathway to reduce microglial activation and decrease the release of proinflammatory factors; simultaneously, it inhibited the activation of the C-X-C motif chemokine ligand 10/C-X-C motif chemokine receptor 3 axis to alleviate central sensitization. These findings suggest that early HF-SCS intervention can improve the functional prognosis after SCI by suppressing neuroinflammation and reducing central sensitization, thereby providing a theoretical basis for the treatment of SCI with HF-SCS.

## Introduction

Spinal cord injury (SCI) is a severe neurological disorder characterized primarily by permanent sensory and motor dysfunction (1). Up to 53% of patients with SCI develop neuropathic pain (NP), a common and refractory complication (2). The typical clinical manifestations of NP include spontaneous burning pain, stabbing pain and electric shock sensations, as well as hyperalgesia and allodynia (3). In addition to sensory symptoms, post-SCI NP markedly impairs the ability of patients to perform daily living activities, restricts social participation and significantly reduces their overall quality of life.

At present, the pharmacological management of post-SCI NP poses substantial challenges. Existing medications, such as anticonvulsants and tricyclic antidepressants, exhibit limited efficacy and are frequently associated with notable adverse effects, including cognitive impairment (such as forgetfulness), somnolence, worsening of spasticity and treatment discontinuation due to intolerability (4). Although opioids provide certain analgesic benefits, their long-term use involves risks of tolerance and dependence (5). Therefore, a reliance solely on pharmacotherapy seldom results in durable and satisfactory pain control. An urgent need exists to adopt non-pharmacological interventions as alternatives or adjuncts to conventional drug therapy to improve the prognosis of patients with post-SCI NP.

The pathogenesis of post-SCI NP is complex, with secondary neuroinflammatory cascades playing a central role in its development and maintenance (6). As resident immune cells of the central nervous system, microglia are rapidly activated following SCI and become the primary drivers of neuroinflammation (7). Activated microglia produce large amounts of reactive oxygen species (ROS), which subsequently activate members of the mitogen-activated protein kinase (MAPK) family, especially p38 MAPK (8). In the context of SCI, p38 MAPK is robustly activated in microglia and its phosphorylation facilitates NF-κB nuclear translocation (8,9). Once activated, NF-κB upregulates the expression of proinflammatory cytokines (such as IL-1β, IL-6 and TNF-α) and other inflammatory mediators (10), thereby driving a coordinated inflammatory response. These mediators, together with ROS, form a self-amplifying positive feedback loop that continuously maintains microglia in an activated state (11), consequently exacerbating central sensitization in the spinal dorsal horn (SDH) and ultimately driving the onset and chronicity of NP (12). In the spinal cord, direct inhibition of the NF-κB signaling pathway can prevent microglia from expressing certain proinflammatory cytokines, thereby alleviating inflammation-induced secondary damage (13). The aforementioned evidence suggests that inhibition of the ROS/p38 MAPK/NF-κB signaling axis is closely associated with reduced microglial activation and the resolution of post-SCI NP.

In addition to the aforementioned classical inflammatory signaling pathways, chemokines also play significant roles in the pathological progression of post-SCI NP (14). C-X-C motif chemokine ligand 10 (CXCL10) is a classic pain-associated chemokine that specifically binds to its receptor, C-X-C motif chemokine receptor 3 (CXCR3). A previous study has shown that CXCR3 is expressed in the dorsal horn neurons of the spinal cord and is significantly upregulated following nerve injury (15); in peripheral nerve injury models, the CXCL10/CXCR3 signaling pathway is associated with the development of hyperalgesia and central sensitization (16). Functional studies have shown that an intraspinal injection of CXCL10 or perfusion of CXCL10 in isolated spinal cord slices can induce hyperalgesia and enhance excitatory synaptic transmission (15,17); conversely, gene knockdown or intrathecal administration of its antagonist significantly alleviates NP symptoms (18). However, systematic studies on the expression patterns of the CXCL10/CXCR3 pathway in post-SCI NP and its association with neuroinflammation are currently lacking.

Spinal cord stimulation (SCS) is a well-established neuromodulation technique that is suitable for pain management when drug therapy is ineffective. Conventional low-frequency SCS relies on inducing paresthesia to exert its analgesic effects; by contrast, 10 kHz high-frequency SCS (HF-SCS) can achieve sustained analgesia without causing paresthesia, has no risk of drug dependence and effectively relieves pain in refractory patients who are unresponsive to conventional SCS (19). Preclinical studies have confirmed that SCS exerts analgesic and neuroprotective effects through multiple mechanisms (20,21). However, conventional SCS protocols may actually increase M1-like microglial activity, thereby attenuating their analgesic effects (22). In spared nerve injury models, HF-SCS treatment has been shown to attenuate NP by suppressing the phosphorylation of ERK, JNK and p38 in the dorsal root ganglia (DRG) and SDH (23), with the concomitant inhibition of microglial activation (24,25). Nevertheless, whether HF-SCS can therapeutically engage the ROS/p38 MAPK/NF-κB and CXCL10/CXCR3 pathways to suppress post-SCI NP remains unexplored. A previous study using 1,200 Hz SCS in a rat thoracic contusion model revealed only mild and transient analgesic effects, suggesting that the optimal stimulation parameters and underlying mechanisms for SCI-specific applications require further investigation (26).

The present study explored the hypothesis that HF-SCS can alleviate microglial activation by inhibiting the p38 MAPK/NF-κB and CXCL10/CXCR3 pathways, thereby alleviating NP and promoting functional recovery in SCI rats.

## Materials and methods

### Animals

In total, 69 (8 weeks old, weighing 220-240 g) female Sprague-Dawley rats were obtained from SPF (Beijing) Biotechnology Co., Ltd. [production license: SCXK (Jing) 2024-0001] and housed in the Animal Laboratory at the China Rehabilitation Research Center (Beijing, China). Only female rats were included in the present study to eliminate potential sex-related variations and reduce the risk of urinary tract infections (27). The rats were housed under controlled environmental conditions (temperature, 22±2°C; humidity, 50±10%) with a 12-h light/dark cycle, with 3 rats per cage. Prior to the commencement of the experimental procedures, the rats were allowed to acclimate to their surroundings for 1 week. The rats were randomly divided into four groups: i) Sham (laminectomy only; n=18); ii) SCI (T10 contusion; n=18); iii) SCI + Sham-stim (electrode implantation without stimulation; n=15); and iv) SCI + SCS (electrode implantation with stimulation; n=18). The sample size of the SCI + Sham-stim group was smaller than that of the other groups, as RNA sequencing (RNA-seq) was not performed on this group. This study was approved by the Animal Experiments and Experimental Animal Welfare Committee of Capital Medical University (Beijing, China) on January 7, 2026 (approval no. AEEI-2026-07). All procedures were performed according to the guidelines stipulated by the Animal Research: Reporting of *in vivo* Experiments (28).

### SCI model and electrode implantation

**Figure 1:**
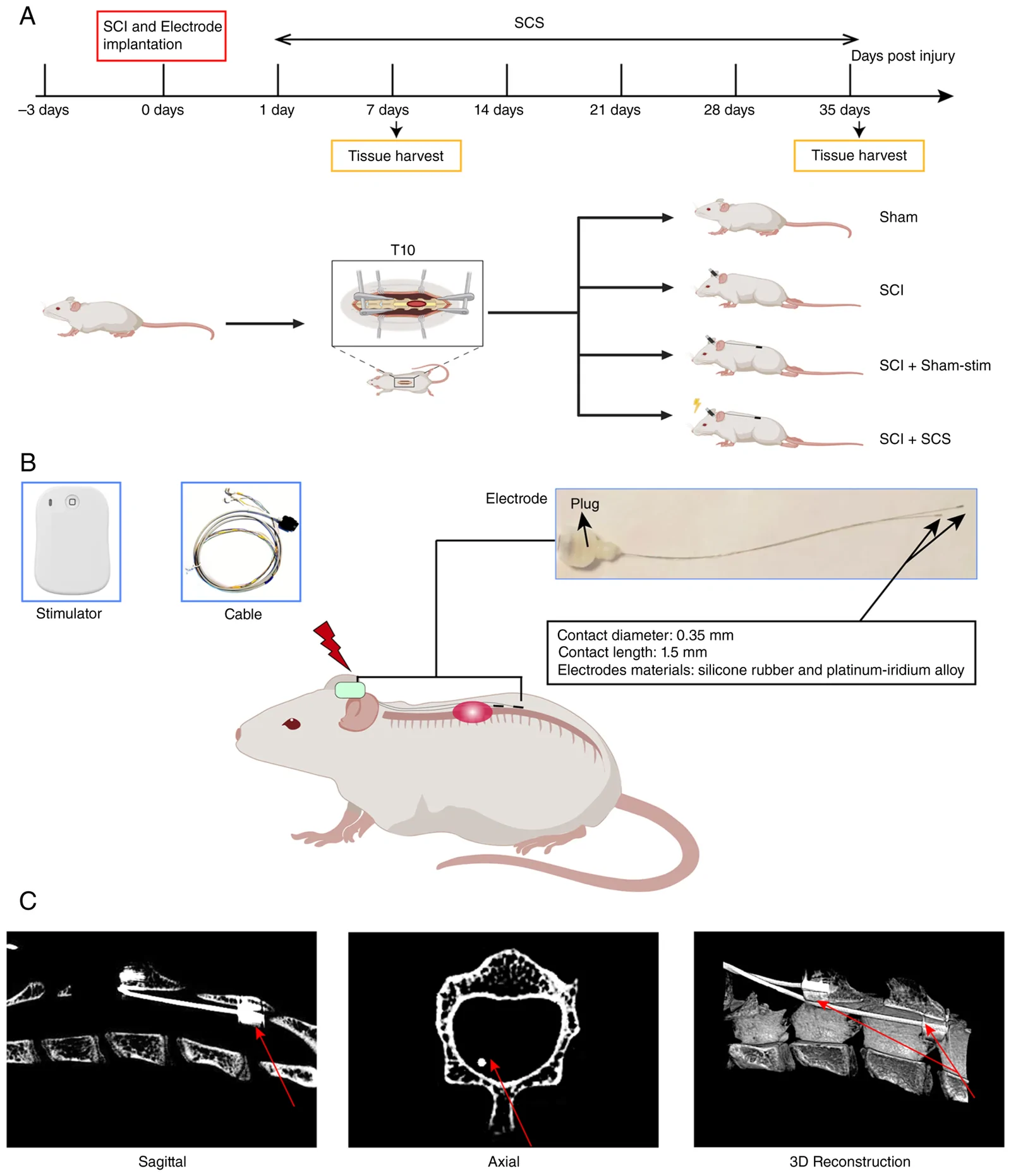
Experimental design and setup for SCS in a rodent SCI model. (A) Timeline of the experimental procedures and group allocation. (B) Schematic of the SCS system comprising a stimulator, connecting cable and silicone rubber electrode with platinum-iridium alloy contacts (0.35 mm contact diameter, 1.5 mm contact length). (C) Representative microcomputed tomography images (axial, sagittal and 3D reconstruction views) confirming electrode placement at the T11-T12 vertebral level (red arrows). SCI, spinal cord injury; SCS, spinal cord stimulation; d, days. Created in BioRender. Liu, T. (2026) https://BioRender.com/7nxzm3q.

The surgical procedures for establishing the SCI model were performed as previously described (29,30). Briefly, the rats were anesthetized with a combination of isoflurane (RWD Life Science Co., Ltd.; cat. no. R510-22-10) and air (4% isoflurane for induction and 2% for maintenance). Laminectomy was performed at T10 to expose the spinal cord, followed by a contusion injury (150 kdynes) using an Infinite Horizon Spinal Cord Impactor (model no. IH-400; Precision Systems and Instrumentation). Successful contusion was confirmed by immediate spinal cord congestion and bilateral hindlimb paralysis. After the T10 contusion injury, a bipolar platinum-iridium electrode (model no. 202S-80; Rishena Medical Co., Ltd.) was implanted into the epidural space through the T10 laminectomy site. The detailed specifications of the electrode are illustrated in Fig. 1B. The electrode was advanced caudally so that the stimulating contacts were positioned at the T11-T12 vertebral level. The electrodes were fixed to the paraspinal ligaments and muscles with sutures. The cathode and anode leads were tunneled subcutaneously to the dorsal cervical region, where the plug was externalized and securely affixed to the skin with dental cement (Shanghai Yuyan Instruments Co., Ltd.). In the SCI group, an electrode plug (without an implanted electrode) was fixed similarly to the neck skin surface with dental cement to maintain an identical external appearance and enable a blinded behavioral assessment. The wound was then sutured layer by layer, and the animals were kept warm until they were fully awake. In the Sham group, an identical surgical exposure was performed without spinal cord contusion or electrode implantation. Postoperatively, the bladders were manually expressed twice daily until spontaneous voiding resumed.

### Stimulation protocol

During stimulation, the conductive cable was connected to both the electrode plug and the stimulator (model no. SCS311; Rishena Medical Co., Ltd.; Fig. 1B), and the stimulation parameters were set using a Bluetooth programmer. The motor threshold (MT) was determined according to previously described methods (31). These rats received direct current square wave pulse stimulation (frequency 2 Hz, pulse width 200 *μ*sec) while awake; the current intensity was gradually increased until symmetrical contractions were observed or palpated in the lower trunk and/or hind limbs for the first time; the current intensity at this time was defined as the MT. Based on a previous study, 40% of the MT intensity was selected as the stimulation intensity (24). In the present study, a 40% MT corresponded to 0.12 mA. HF-SCS was initiated at 1 day post-injury (dpi) and was delivered for 2 h daily through 35 dpi (10 kHz, 0.12 mA, 30 *μ*sec pulse width). Specifically, previous studies have shown that 10 kHz SCS at a 40% MT consistently inhibits mechanical hypersensitivity without inducing motor responses or behavioral signs of discomfort in rats with a nerve injury (32,33). The rats in the SCI + Sham-stim group underwent identical electrode implantation procedures without current delivery.

### Mechanical and thermal hypersensitivity

The paw withdrawal mechanical threshold (PWMT) was assessed using von Frey filaments (cat. no. 58011; Stoelting Co.) with the up-down method (34). Prior to testing, the rats were acclimated to the experimental environment for 30 min. The test was initiated with a 2 g filament, which was applied perpendicularly to the plantar surface of the rat's hind paw. A positive response (licking or withdrawal) was followed by a thinner filament and a negative response was followed by a thicker filament. Testing continued for six applications or until four consecutive positive or negative responses were obtained. Finally, the 50% PWMT (in grams) was calculated. Only rats with 50% PWMT <4 g were selected and used for subsequent experiments.

The paw withdrawal latency (PWTL) was assessed using an infrared thermal stimulator (RWD Life Science Co., Ltd.) as previously described (35). The rats were acclimated for 30 min in a Plexiglas chamber on a glass stand. A radiant heat source (50% IR intensity) was applied to the plantar hind paw, and the withdrawal latency was automatically recorded. A 25 sec cut-off was used to prevent tissue damage. Each paw was tested in triplicate at 5 min intervals and the values were averaged. All behavioral assessments were conducted by two independent investigators who were blinded to the group allocation.

### Basso, Beattie and Bresnahan scale scores and the inclined plane test

The recovery of motor function was evaluated using the Basso, Beattie and Bresnahan (BBB) locomotor scale (ranging from 21 points for normal function to 0 points for complete paralysis) (36) and the inclined plane test at baseline and at 1, 7, 14, 21, 28 and 35 dpi. Hindlimb strength was assessed using the inclined plane test, which was performed as previously described (30). The tests were conducted by two independent investigators who were blinded to the group allocation.

### Open field test

The open field test was performed to assess locomotor function and anxiety levels (37). In the open field test, each rat was placed in the center of a 40×40 cm square arena (TAP Plastics, Inc.) and monitored in a 5-min session; locomotor activity was recorded by an overhead webcam connected to a laptop. A CleverSys TopScan Lite device (CleverSys, Inc.) was used to automatically track and analyze the movements of the animals, including the total distance traveled during the session and the number of times the rats crossed the central area.

### Gait analysis

A gait analysis was performed at 35 dpi. Gait data were quantified using the DigiGait Imaging System (Mouse Specifics, Inc.). DigiGait automatically identified and analyzed the gait patterns of the rats while they were walking on a treadmill. In the present study, the treadmill speed was set at 10 cm/sec, and the following dynamic gait indices were calculated using DigiGait analysis software version 5.0 (Mouse Specifics, Inc.): paw area, stride length and gait symmetry. Gait symmetry values closer to 1 indicate improved coordination between the forelimbs and hindlimbs (38,39).

### Monitoring behaviors in the home cage

Animals were individually housed in transparent cages equipped with food/water stations and bedding within a barrier facility with controlled ventilation and lighting to minimize external disturbances. A 2-h video recording session was conducted starting at 9:00 AM, and the footage was analyzed using HomeCageScan software version 3.0 (CleverSys, Inc.). The behavioral data are reported as the percentage of time spent on each activity. Rearing, grooming and jumping represent exploration, self-care and voluntary locomotion, respectively (40). Excessive grooming is considered an indicator of pain-related behavior (41).

### Electrophysiological recording

At 35 dpi, motor evoked potentials (MEPs) were recorded using an electrophysiological system (Zhuhai Iridium Nanotechnology Co., Ltd.) under isoflurane anesthesia (4% isoflurane for induction and 2% for maintenance), as described previously (30). Briefly, the stimulating electrode was placed subcutaneously above the anterior fontanelle, and the recording electrode was inserted into the Achilles tendon. Epidural stimulation was suspended for at least 10 min prior to recording. Each measurement was repeated three times per animal. The amplitude and latency were analyzed to evaluate motor functional recovery.

### Microcomputed tomography (micro-CT)

Micro-CT scanning was performed *ex vivo* on harvested spinal column specimens after animal sacrifice, using a Quantum FX system (PerkinElmer, Inc.) to verify the electrode placement. Multiplanar reconstructed images (sagittal, axial and 3D) were generated using Analyze software version 12.0 (Caliper Life Sciences; PerkinElmer, Inc.) to visualize the spatial relationship between the electrodes and vertebral structures.

### Tissue preparation

At 7 dpi, the injured spinal cord was harvested for the acute phase analysis. At 35 dpi, three regions were collected: i) The injured spinal cord; ii) the SDH (L4-L6); and iii) the bilateral L4-L6 DRG. Rats were euthanized by an overdose of isoflurane (5% isoflurane in room air until breathing ceased) and death was confirmed by the absence of a heartbeat and the loss of corneal and pedal withdrawal reflexes prior to perfusion. For histology and immunofluorescence staining, rats were transcardially perfused with 0.9% NaCl followed by 4% paraformaldehyde (PFA). The tissues were postfixed with 4% PFA for 24 h at 4°C, cryoprotected in 20 and 30% sucrose and sectioned at 10 *μ*m using a cryostat (Leica Microsystems GmbH; model no. CM1950). Sections were mounted on adhesive slides, air-dried for 30 min and stored at −20°C. For Western blotting and RNA extraction, additional rats were perfused with 0.9% NaCl only; the SCI site and DRG were rapidly dissected, snap-frozen in liquid nitrogen and stored at −80°C.

### RNA-seq

Total RNA was extracted from the T9-T11 spinal cord segments of rats in the Sham, SCI and SCI + SCS groups using TRIzol reagent (cat. no. 15596018; Invitrogen; Thermo Fisher Scientific, Inc.). RNA concentration was measured using the Qubit RNA BR (Broad-Range) Assay Kit (cat. no. Q10210; Invitrogen; Thermo Fisher Scientific, Inc.) on a Qubit 3 Fluorometer (Thermo Fisher Scientific, Inc.), and RNA purity was assessed using a NanoDrop One spectrophotometer (model ND-ONE-W; Thermo Fisher Scientific, Inc.). RNA integrity was assessed using an Agilent 4150 TapeStation (Agilent Technologies, Inc.). The RNA-seq library was prepared by Wuhan Servicebio Technology Co., Ltd. Briefly, mRNA was enriched using oligo(dT) magnetic beads with the Hieff NGS^®^ mRNA Isolation Master Kit V2 (cat. no. 12629ES96; Shanghai Yeasen Biotechnology Co., Ltd.). The enriched mRNA was fragmented and reverse transcribed into cDNA. Library construction was performed using the Hieff NGS^®^ EvoMax RNA Library Prep Kit (dNTP) (cat. no. 12341ES97; Shanghai Yeasen Biotechnology Co., Ltd.). After end repair, adapter ligation and PCR amplification, the quality of the resulting libraries was verified by measuring the concentration using the Equalbit 1X dsDNA HS Assay Kit (cat. no. EQ121-02; Vazyme Biotech Co., Ltd.) and assessing the fragment size distribution using the Agilent 4200 TapeStation System (Agilent Technologies, Inc.) with D1000 Screen Tape (cat. no. 5067-5582; Agilent Technologies, Inc.). The resulting libraries were circularized into single-stranded circular libraries using the Hieff NGS^®^ Dual Barcode Fast-Pace DNA Cyclization Kit for MGI (cat. no. 13340ES96; Shanghai Yeasen Biotechnology Co., Ltd.) and subsequently quantified using the Equalbit™ ssDNA Assay Kit (cat. no. EQ311-02; Vazyme Biotech Co., Ltd.) on a Qubit 3 Fluorometer (Thermo Fisher Scientific, Inc.). The final loading concentration of the DNB libraries was 4 nM. The circularized libraries were converted into DNA nanoballs and loaded onto the DNBSEQ-T7RS sequencing platform (MGI Tech Co., Ltd.) using the DNBSEQ-T7RS High-Throughput Sequencing Reagent Kit (FCL PE150) V3.0 (cat. no. 940-000266-00; MGI Tech Co., Ltd.) for paired-end 150 bp sequencing. Raw reads were processed using fastp (version 0.23.2; OpenGene) to remove adapter sequences and low-quality reads. Differentially expressed genes (DEGs) were identified using DESeq2 (version 1.42.1; Bioconductor) with a |log_2_ fold change (FC)|>1 and an adjusted P<0.05. The functional enrichment analysis, protein-protein interaction (PPI) network analysis and gene set enrichment analysis (GSEA) were performed using clusterProfiler (version 4.10.1; Bioconductor;), STRINGdb (version 12.0; https://string-db.org/) and Gsea (version 4.3.3; Broad Institute), respectively. Bubble plots of enriched Kyoto Encyclopedia of Genes and Genomes (KEGG) pathways were generated using the Weishengxin online platform (www.bioinformatics.com.cn). RNA-seq datasets were deposited in the National Center for Biotechnology Information Gene Expression Omnibus database (ncbi.nlm.nih.gov/geo; accession no. GSE332999).

### Hematoxylin-eosin (HE) and Nissl staining

For HE staining, frozen sections were thawed at room temperature for 30 min. The nuclei were stained with a hematoxylin solution (Beijing Solarbio Science & Technology Co., Ltd.) for 5 min at 20-25°C, followed by differentiation in 1% acid alcohol for 30 sec. The sections were then rinsed with tap water for 10 min, counterstained with an eosin solution for 3 min at 20-25°C and dehydrated through a graded ethanol series. After clearing in xylene (two changes, 5 min each), the sections were permanently mounted with neutral resin and coverslipped for examination under a light microscope.

For Nissl staining, the sections were thawed as aforementioned. The sections were stained with Nissl solution (Wuhan Servicebio Technology Co., Ltd.) at 37°C for 10-15 min and briefly differentiated in 0.1% glacial acetic acid (~30 sec). The sections were then rinsed with distilled water and air-dried at room temperature for 2 h. Finally, the sections were coverslipped with neutral resin for light microscopic analysis.

### Immunofluorescence staining

The frozen sections were thawed at room temperature, permeabilized with 0.5% Triton X-100 for 10 min, blocked with 2% BSA (Thermo Fisher Scientific, Inc.) for 1 h at room temperature, incubated with primary antibodies overnight at 4°C and then with CoraLite-conjugated secondary antibodies for 1 h at room temperature. After 4',6-diamidino-2-phenylindole (DAPI) counterstaining for 10 min at room temperature, whole-slide images were acquired using the TissueFAXS PLUS system and analyzed with ImageJ 1.54p software (National Institutes of Health) to determine colocalization and the fluorescence intensity. The primary antibodies used were rabbit anti-CXCR3 (1:200; Affinity Biosciences; cat. no. DF7113), rabbit anti-CXCL10 (1:200; Affinity Biosciences; cat. no. DF6417), mouse anti-ionized calcium-binding adapter molecule 1 (Iba-1; 1:200; Abcam; cat. no. ab283319), rabbit anti-phosphorylated (p-)p65 (Ser536) (1:500; CST Biological Reagents Co., Ltd.; cat. no. 3033), rabbit anti-p-p38 (Thr180/Tyr182) (1:800; CST Biological Reagents Co., Ltd.; cat. no. 4511), mouse anti-β-III tubulin (Tuj1; 1:1,000; Abcam; cat. no. ab78078), chicken anti-glial fibrillary acidic protein (GFAP; 1:200; Abcam; cat. no. ab4674), mouse anti-neurofilament 200 (NF200; 1:400; CST Biological Reagents Co., Ltd.; cat. no. 2836), rabbit anti-myelin basic protein (MBP; 1:50; CST Biological Reagents Co., Ltd.; cat. no. 78896) and rabbit anti-c-Fos (1:500; CST Biological Reagents Co., Ltd.; cat. no. 2250). The secondary antibodies used were CoraLite 488-conjugated goat anti-rabbit IgG (H+L) (1:500; Proteintech Group, Inc.; cat. no. SA00013-2), CoraLite 488-conjugated goat anti-mouse IgG (H+L) (1:500; Proteintech Group, Inc.; cat. no. SA00013-1), CoraLite 594-conjugated goat anti-rabbit IgG (H+L) (1:500; Proteintech Group, Inc.; cat. no. SA00013-4), CoraLite 594-conjugated goat anti-mouse IgG (H+L) (1:500; Proteintech Group, Inc.; cat. no. SA00013-3) and goat anti-chicken IgY H&L (Alexa Fluor^®^ 488) (1:500; Abcam; cat. no. ab150169).

### Dihydroethidium (DHE) staining

Oxidative stress was evaluated by performing DHE staining according to a previously published protocol (42). Briefly, frozen spinal cord sections were incubated with the fluorescent dye DHE (Beyotime Biotechnology; cat. no. S0063). After three washes with PBS, the stained sections were observed using the TissueFAXS PLUS system, and the fluorescence intensity was quantified using ImageJ software.

### Western blot analysis

The tissue samples were homogenized in RIPA lysis buffer (Beyotime Biotechnology) containing protease and phosphatase inhibitors on ice. After centrifugation at 12,000 × g for 15 min at 4°C, the protein concentration was determined using the BCA method (Wuhan Servicebio Technology Co., Ltd.). Equal amounts of protein (20 *μ*g/lane) were denatured and separated by 10% SDS-PAGE and then transferred to PVDF membranes (Wuhan Servicebio Technology Co., Ltd.). After blocking with 5% non-fat milk for 1 h at room temperature, the membranes were incubated with the following primary antibodies overnight at 4°C: Anti-CXCR3 (1:1,000; Affinity Biosciences; cat. no. DF7113), anti-CXCL10 (1:1,000; Affinity Biosciences; cat. no. DF6417), anti-p-p65 (1:1,000; CST Biological Reagents Co., Ltd.; cat. no. 3033), anti-p-p38 (1:1,000; CST Biological Reagents Co., Ltd.; cat. no. 4511), anti-p65 (1:1,000; CST Biological Reagents Co., Ltd.; cat. no. 8242), anti-p38 (1:1,000; CST Biological Reagents Co., Ltd.; cat. no. 8690), anti-TNF-α (1:1,000; Abmart Pharmaceutical Technology Co., Ltd.; cat. no. PY19810) and anti-IL-1β (1:1,000; ImmunoWay Biotechnology Company; cat. no. YM8498) (all rabbit). After washing with TBST (0.1% Tween), the membranes were incubated with an HRP-conjugated goat anti-rabbit IgG (H+L) secondary antibody (1:5,000; Wuhan Servicebio Technology Co., Ltd.; GB23303) for 30 min at room temperature. The protein bands were detected using enhanced chemiluminescence reagents (cat. no. G2020; Wuhan Servicebio Technology Co., Ltd.) and semi-quantified by densitometric analysis with ImageJ software.

### Statistical analysis

Data are presented as the mean ± standard deviation. Comparisons among multiple groups were performed using one-way analysis of variance (ANOVA) with Tukey's post hoc test. Longitudinal functional assessments (PWMT, PWTL, BBB scores and inclined plane test) were performed using two-way repeated-measures ANOVA followed by Tukey's post hoc test to examine differences between groups at each time point. Statistical analyses were conducted using GraphPad Prism 9.0 (Dotmatics). P<0.05 was considered to indicate a statistically significant difference.

## Results

### HF-SCS attenuates NP and improves functional recovery after SCI

**Figure 2:**
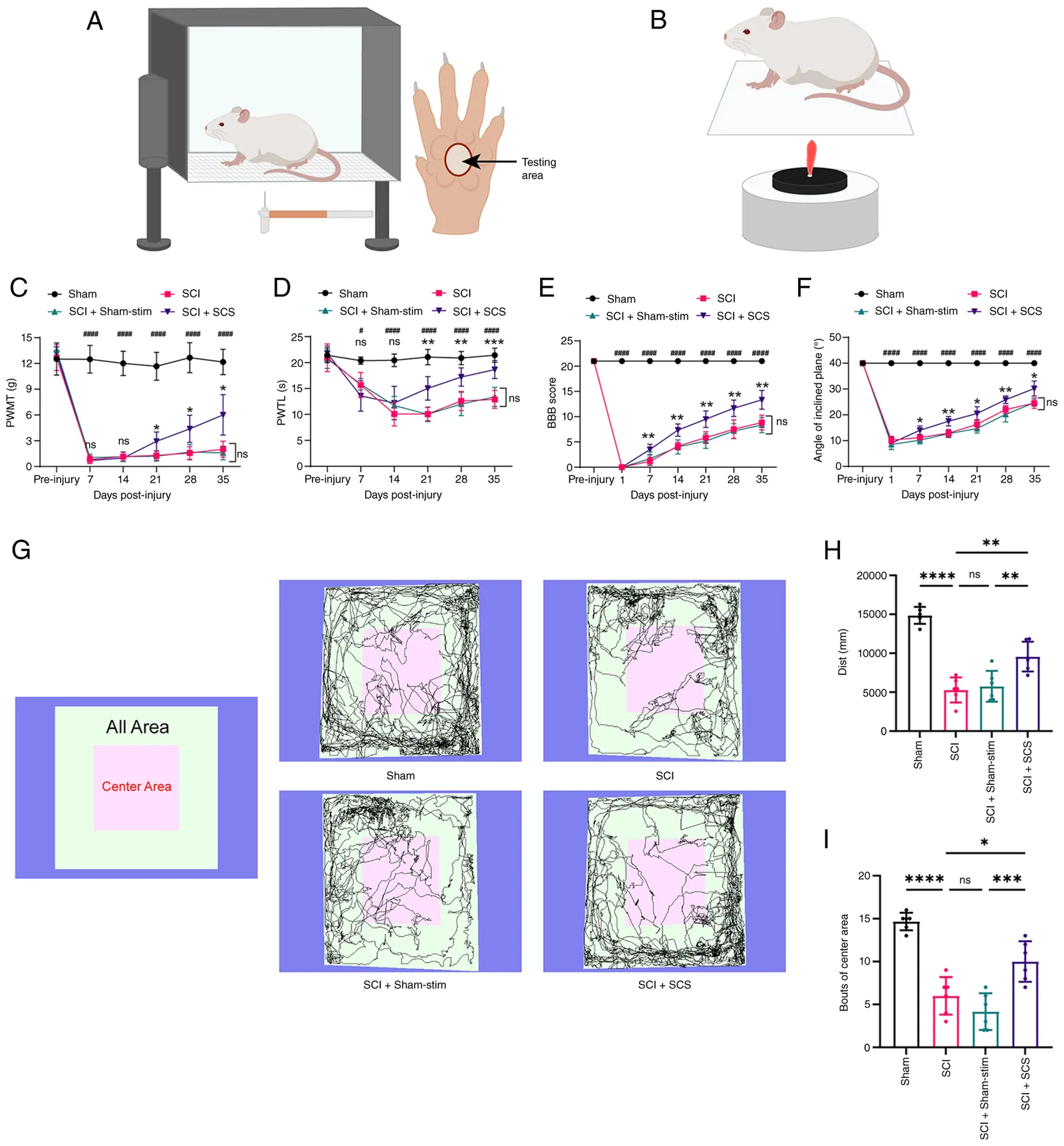
HF-SCS alleviates neuropathic pain and enhances motor function recovery after SCI. (A) Diagram of the von Frey test. (B) Diagram of the thermal hyperalgesia test. (C) Mechanical allodynia evaluated by the PWMT at baseline and 7, 14, 21, 28 and 35 dpi. (D) Thermal hyperalgesia evaluated by the PWTL at baseline and 7, 14, 21, 28 and 35 dpi. (E) The BBB score and (F) inclined plane test results were used to evaluate the recovery of motor function in each group at baseline and at 1, 7, 14, 21, 28 and 35 dpi. (G) Representative tracks and schematic of the open field test. Quantification of the (H) total distance traveled and (I) number of center entries from the open field test at 35 dpi. The data are presented as the mean ± SD (n=6). ^*^P<0.05, ^**^P<0.01, ^***^P<0.001, ^****^P<0.0001 for the comparison of the SCI + SCS and SCI groups; ^#^P<0.05, ^####^P<0.0001 for the comparison of the SCI and Sham groups; two-way repeated-measures analysis of variance followed by Tukey's post hoc test (C-F) or one-way analysis of variance followed by Tukey's post hoc test (H and I). ns, not significant; PWMT, paw withdrawal mechanical threshold; PWTL, paw withdrawal latency; dpi, days post-injury; BBB, Basso, Beattie and Bresnahan; SCI, spinal cord injury; HF-SCS, high-frequency spinal cord stimulation.

The experimental design of the present study is outlined in Fig. 1A. Micro-CT scanning was performed to verify the precise anatomical placement of the electrodes at the T11-T12 vertebral level (Fig. 1C). The PWMT and PWTL were evaluated to investigate the therapeutic effect of HF-SCS on post-SCI NP (Fig. 2A-D). At 7 dpi, compared with the Sham group, all three SCI surgery groups displayed significantly lower PWMT (P<0.0001) and PWTL (P<0.05), confirming the successful establishment of the post-SCI NP rat model. At 21, 28 and 35 dpi, the PWMT of the SCI + SCS group was significantly improved compared with that of the SCI group (P<0.05), whereas no significant difference was observed between the SCI + Sham-stim and SCI groups (P>0.05). Compared with the SCI group, the SCI + SCS group had a significantly improved PWTL throughout the experiment (P<0.05), whereas no significant difference was observed between the SCI + Sham-stim and SCI groups (P>0.05). These data indicate that HF-SCS treatment effectively alleviated mechanical allodynia and thermal hyperalgesia in SCI rats.

The BBB scale score was determined and inclined plane test (maximum angle maintained) was performed to assess motor function. The BBB scores of the SCI + SCS group were significantly improved compared with those of the SCI group (P<0.01; Fig. 2E). The results of the inclined plane test further corroborated these findings; the maximum inclination angle was markedly increased in the SCI + SCS group (P<0.05), whereas no significant difference was observed between the SCI + Sham-stim and SCI groups (P>0.05) (Fig. 2F).

**Figure 3:**
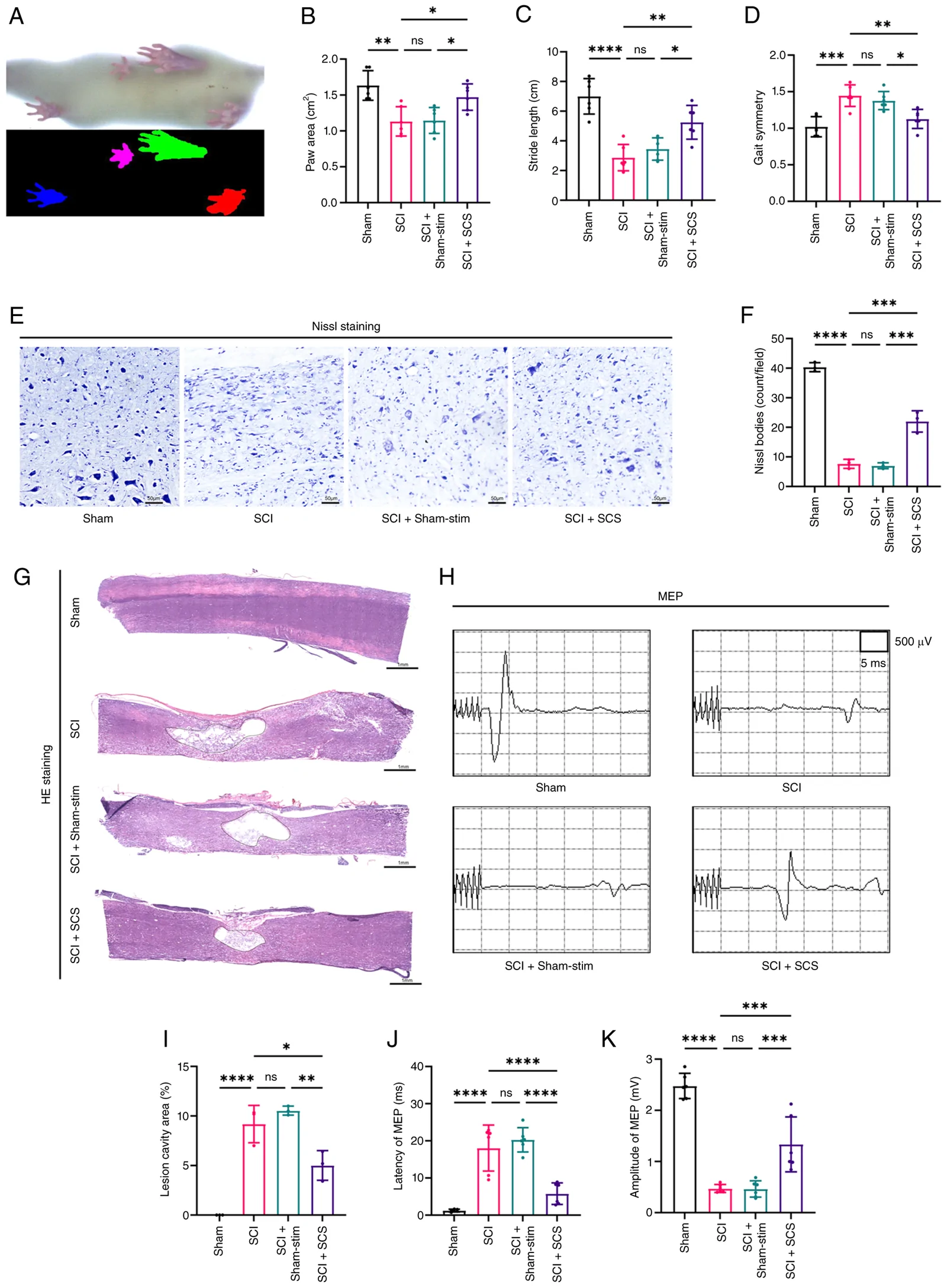
HF-SCS improves gait performance, tissue repair and functional recovery in SCI rats. (A) Schematic illustration of the DigiGait gait analysis system. Quantitative gait analysis at 35 dpi showing (B) the paw contact area, (C) stride length and (D) gait symmetry index (n=6). Nissl staining of the spinal cord surrounding the injury core at 35 dpi: (E) representative images and (F) quantification of the number of Nissl bodies (scale bar, 50 *μ*m; n=3). (G) Representative images of HE staining (scale bar, 1 mm; n=3). (H) Representative MEP waveforms demonstrating electrophysiological recovery. (I) Quantification of the lesion cavity area. Quantitative analysis of MEP parameters indicating (J) reduced latency and (K) increased amplitude at 35 dpi (n=6). The data are presented as the mean ± SD. ^*^P<0.05, ^**^P<0.01, ^***^P<0.001, ^****^P<0.0001; one-way analysis of variance, followed by Tukey's post hoc test. ns, not significant; dpi, days post-injury; HE, hematoxylin-eosin; HF-SCS, high-frequency spinal cord stimulation; MEPs, motor evoked potentials; SCI, spinal cord injury.

A number of behavioral assessments, including open field testing, gait analysis, automated home cage monitoring and electrophysiological recordings, were conducted at 35 dpi to comprehensively assess functional recovery following SCI. In the open field test, the SCI + SCS group exhibited a significantly greater total distance traveled (P<0.01) and more center area entries (P<0.05) than the SCI group (Fig. 2G-I). Additionally, the DigiGait analysis revealed an increased paw contact area (P<0.05), stride length (P<0.01) (Fig. 3A-C), indicating improved weight-bearing capacity and locomotor function. Regarding gait symmetry, compared with that in the Sham group, SCI significantly increased the gait symmetry index and HF-SCS treatment significantly alleviated this alteration (P<0.01; Fig. 3D), reflecting restored gait coordination. Automated home cage monitoring further showed that, compared with the SCI group and the SCI + Sham-stim group, the numbers of rearing and jumping behaviors were increased in rats from the SCI+SCS group (P<0.05; Fig. S1B and D), whereas grooming behavior was decreased (P<0.05; Fig. S1C), indicating that HF-SCS facilitated motor recovery while alleviating spontaneous neuropathic pain, consistent with excessive grooming being a recognized marker of pain-related behavior in rodents (41). Electrophysiological recordings showed that the SCI group exhibited a significantly reduced MEP amplitude and prolonged latency compared with the Sham group (P<0.05). Following HF-SCS treatment, the amplitude was significantly increased and the latency was markedly shortened compared with the values in both the SCI and SCI + Sham-stim groups (P<0.05) (Fig. 3H, J and K), suggesting that HF-SCS improved nerve conduction function after SCI.

### HF-SCS alleviates pathological damage and preserves neuronal integrity after SCI

Spinal cord tissue integrity and neuronal survival were evaluated at 35 dpi using HE and Nissl staining, respectively. Quantitative analysis revealed that the SCI group displayed a significantly larger lesion cavity area and more severe neuronal loss compared with the Sham group (P<0.05). By contrast, the SCI + SCS group showed a markedly reduced lesion cavity area and significantly improved neuronal survival relative to both the SCI and SCI + Sham-stim groups (P<0.05) (Fig. 3E-G and I), suggesting that HF-SCS attenuated tissue damage and promoted neuronal preservation following SCI. No significant difference was observed between the SCI and SCI + Sham-stim groups (P>0.05), indicating that electrode implantation did not cause additional damage to the spinal cord tissue and confirming the safety of the implantation procedure. These results indicate that HF-SCS has the potential to mitigate tissue loss and neuronal loss after SCI.

### RNA-seq analysis reveals that HF-SCS modulates neuroinflammatory pathways

**Figure 4:**
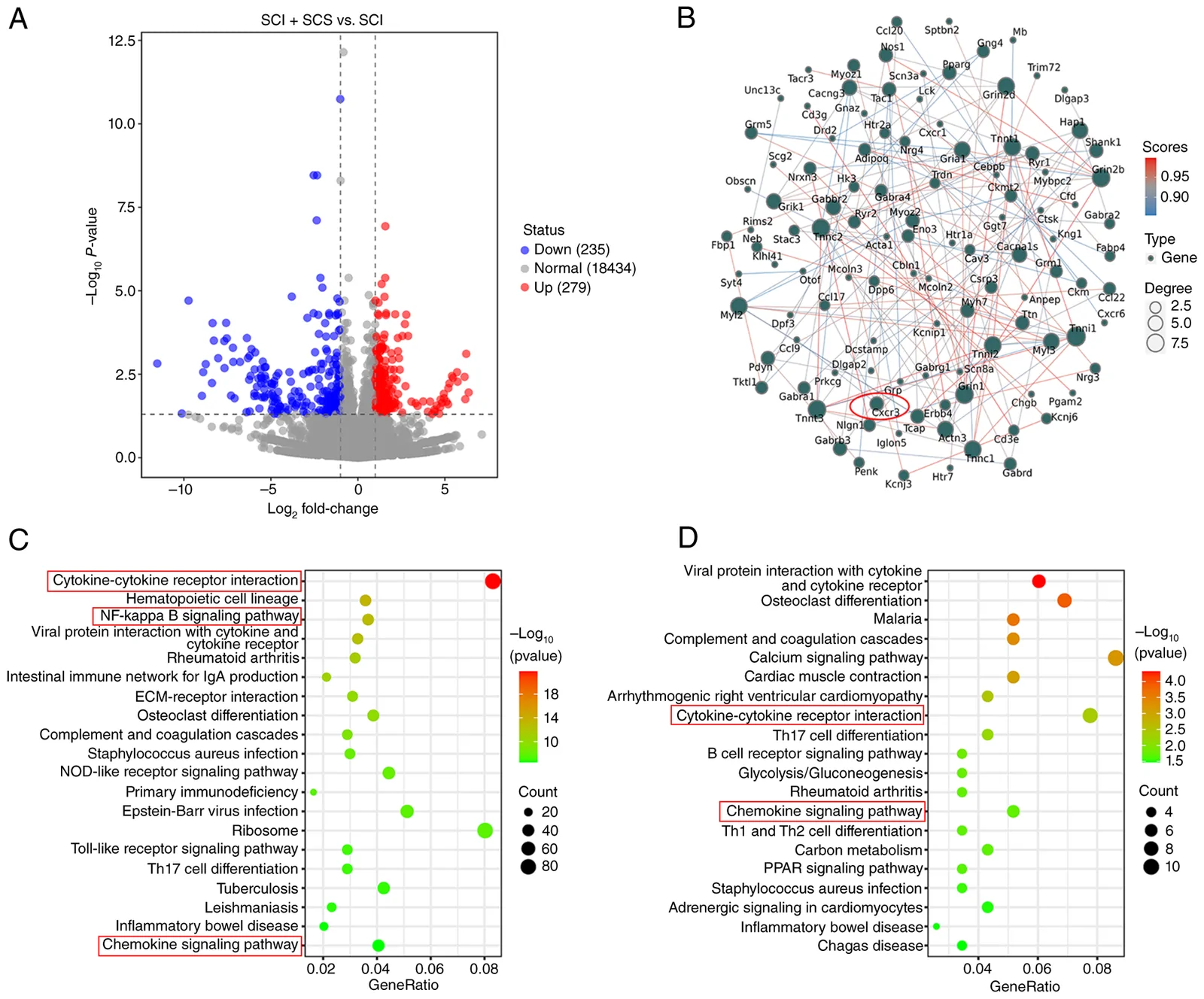
RNA-sequencing analysis of the effect of HF-SCS on SCI rats at 35 dpi. (A) Volcano plot displaying 514 DEGs (279 upregulated genes and 235 downregulated genes) in the SCI + SCS group compared with the SCI group (|log_2_Fold Change|>1, adjusted P<0.05). (B) Protein-protein interaction network analysis of the top-ranked DEGs, with the node size indicating the degree of connectivity and color representing correlation scores. (C) Bubble plot showing upregulated KEGG pathways in the SCI group compared with the Sham group. (D) Bubble plot showing downregulated KEGG pathways in the SCI+SCS group compared with the SCI group (n=3). DEGs, differentially expressed genes; dpi, days post-injury; FDR, false discovery rate; HF-SCS, high-frequency spinal cord stimulation; KEGG, Kyoto Encyclopedia of Genes and Genomes; SCI, spinal cord injury.

Given that NP develops progressively, RNA-seq using spinal cord tissue from the T9-T11 injury epicenter from rats at 35 dpi was performed to capture the sustained transcriptional changes underlying long-term pain relief. This time point was selected to ensure that the observed molecular alterations reflected stable, chronic-phase mechanisms rather than acute injury responses or transient early adaptations. The volcano plot showed that HF-SCS treatment induced 514 DEGs compared with SCI, comprising 279 upregulated and 235 downregulated genes (|log_2_FC|>1, adjusted P<0.05; Fig. 4A). The heatmap further showed distinct gene expression patterns between the SCI and SCI + SCS groups; notably, CXCR3 expression was significantly downregulated following HF-SCS treatment (Fig. S2A). The functional relationships among the DEGs were explored by constructing a PPI network, in which the node size was scaled by connectivity and color-coded by the interaction score. CXCR3 emerged as a key hub gene within this network, suggesting its central role in regulating inflammation following SCI (Fig. 4B). The KEGG enrichment analysis (bubble plot) revealed that 'Cytokine-cytokine receptor interaction', 'NF-κB signaling pathway' and 'Chemokine signaling pathway' were significantly activated after SCI (Fig. 4C), all of which are closely associated with neuroinflammation. HF-SCS treatment appeared to downregulate these pathways, suggesting the potential attenuation of inflammatory signal transduction (Fig. 4D). GSEA further indicated that 'Cytokine-Cytokine Receptor Interaction', 'NF-κB Signaling Pathway' and 'Chemokine Signaling Pathway' were significantly enriched in the SCI group (Fig. S2B-D), whereas HF-SCS treatment suppressed the activity of these pathways (Fig. S2E and F). Collectively, these transcriptomic findings suggest that HF-SCS may exert therapeutic effects potentially by modulating neuroinflammatory signaling pathways.

### HF-SCS attenuates oxidative stress and microglial activation at the acute phase of SCI

**Figure 5:**
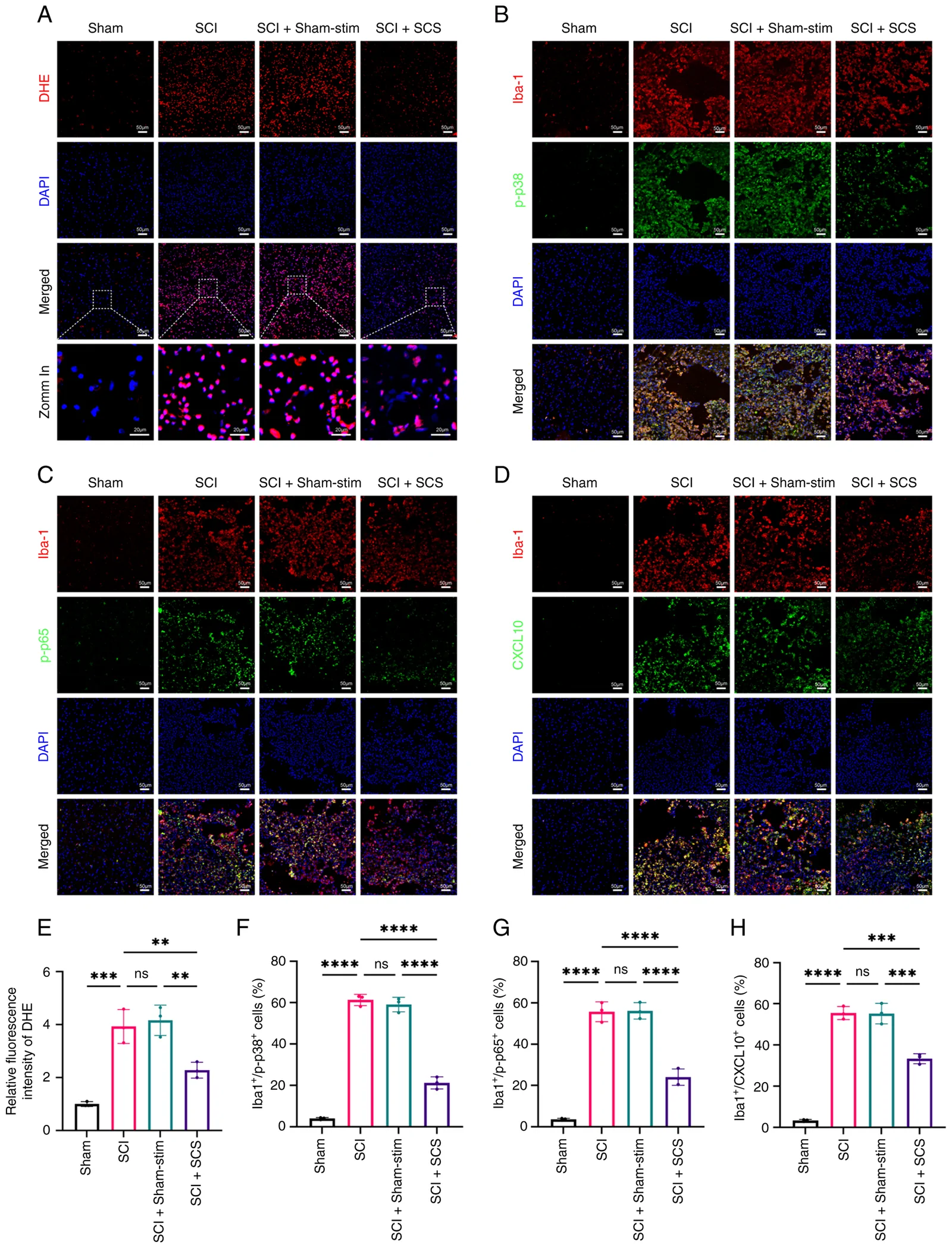
HF-SCS attenuates oxidative stress and microglial activation at the lesion site at 7 dpi. (A) Representative images of DHE staining (red) showing reactive oxygen species levels (scale bars, 50 and 20 *μ*m). DAPI (blue) indicates nuclei. Representative images of double immunofluorescence staining: (B) Iba-1 (red) and p-p38 (green), (C) Iba-1 (red) and p-p65 (green) and (D) Iba-1 (red) and CXCL10 (green) (scale bar, 50 *μ*m). (E) Quantification of the DHE fluorescence intensity. Quantification of double-positive microglia: (F) p-p38^+^, (G) p-p65^+^ and (H) CXCL10^+^ (reported as a percentage of the total number of Iba-1^+^ cells). The data are presented as the mean ± SD (n=3). ^**^P<0.01, ^***^P<0.001, ^****^P<0.0001; one-way analysis of variance followed by Tukey's post hoc test. ns, not significant; CXCL10, C-X-C motif chemokine ligand 10; DAPI, 4',6-diamidino-2-phenylindole; DHE, dihydroethidium; dpi, days post-injury; HF-SCS, high-frequency spinal cord stimulation; Iba-1, ionized calcium-binding adapter molecule 1; p-, phosphorylated; SCI, spinal cord injury.

Building upon the RNA-seq findings that identified CXCR3 as a key hub gene within the PPI network and revealed the significant enrichment of cytokine-cytokine receptor interactions and NF-κB signaling pathways at 35 dpi, the upstream events that might precede these pathway alterations were next investigated. Given that ROS act as known upstream triggers for both NF-κB activation and cytokine signaling (43), DHE staining at 7 dpi was performed to assess oxidative stress levels, aiming to determine whether the therapeutic effects on these inflammatory pathways are mediated by early ROS suppression (43,44). At this acute phase, the DHE fluorescence intensity in the SCI group was significantly higher than that in the Sham group (P<0.001; Fig. 5A and E), indicating severe oxidative stress at the lesion site. No significant difference was observed between the SCI + Sham-stim and SCI groups (P>0.05), suggesting that electrode implantation did not exacerbate oxidative damage. Notably, ROS generation was significantly decreased following HF-SCS treatment compared with the SCI and SCI + Sham-stim groups (P<0.01; Fig. 5A and E), suggesting that the effects of HF-SCS attenuated oxidative stress after SCI. Accumulating evidence indicates that microglia-mediated neuroinflammation plays a central role in the pathological progression following SCI (7,45). Therefore, double immunofluorescence staining was performed for the microglial biomarker, Iba-1, together with p-p38, p-p65 or CXCL10 at 7 dpi, to investigate microglia-specific inflammatory signaling. Notably, CXCL10 immunoreactivity was detected within the cytoplasm of microglia, suggesting that it was synthesized or accumulated under pathological conditions. The quantitative analysis revealed that the percentages of Iba-1^+^ microglia co-expressing p-p38, p-p65 or CXCL10 were significantly higher in the SCI group than in the Sham group (P<0.05). Compared with the SCI group, the SCS group had markedly lower proportions of double-positive cells, suggesting that the inflammatory activation of microglia was attenuated (P<0.05) (Fig. 5B-D and F-H).

### HF-SCS downregulates CXCL10/CXCR3 expression and suppresses p38 MAPK/NF-κB-mediated proinflammatory signaling

**Figure 6:**
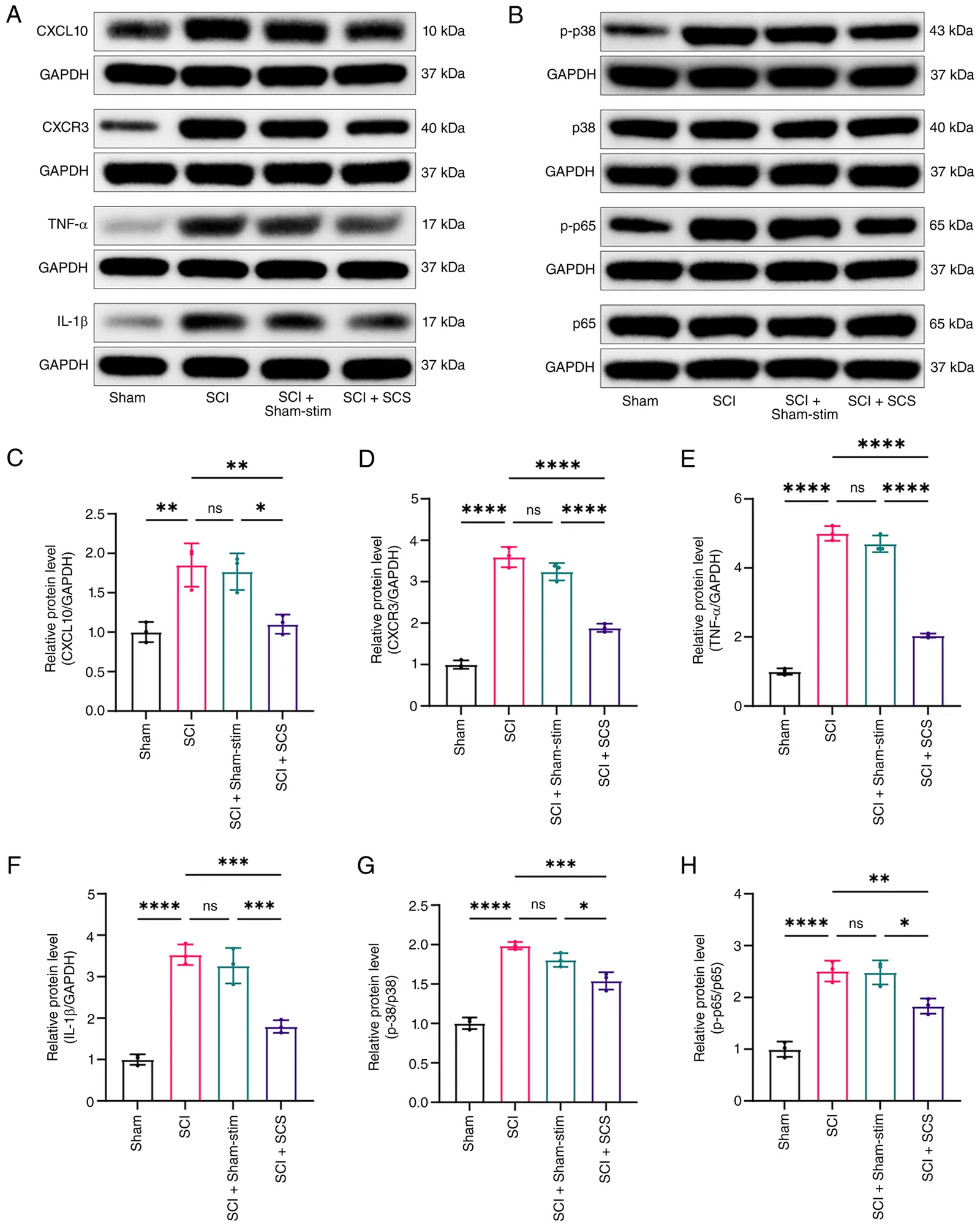
HF-SCS inhibits the activation of the p38 MAPK/NF-κB and CXCL10/CXCR3 pathways at 7 dpi. (A) Representative western blots showing the levels of inflammatory mediators (CXCL10, CXCR3, TNF-α and IL-1β). (B) Representative western blots showing the levels of phosphorylated and total p38 MAPK and NF-κB p65. Quantitative analysis of the expression of (C) CXCL10, (D) CXCR3, (E) TNF-α and (F) IL-1β. Quantitative analysis of the phosphorylation ratios of (G) p-p38/p38 and (H) p-p65/p65. The data are presented as the mean ± SD (n=3). ^*^P<0.05, ^**^P<0.01, ^***^P<0.001, ^****^P<0.0001; one-way analysis of variance followed by Tukey's post hoc test. ns, not significant; HF-SCS, high-frequency spinal cord stimulation; p-, phosphorylated; CXCL10, C-X-C motif chemokine ligand 10; CXCR3, C-X-C motif chemokine receptor 3; dpi, days post-injury; SCI, spinal cord injury.

Western blot analyses were performed on spinal cord tissues at 7 dpi to further validate the results of the immunofluorescence staining and RNA-seq predictions. Consistent with the immunofluorescence staining observations, the protein expression levels of CXCL10 and its cognate receptor, CXCR3, were significantly increased in the SCI group compared with the Sham group, and these increases were significantly attenuated following HF-SCS intervention (P<0.05; Fig. 6A, C and D). The phosphorylation status of p38 and p65 were also examined, the results of which are reported as the ratio of phosphorylated protein to total protein level. The SCI group exhibited significantly higher p-p38/p38 and p-p65/p65 ratios than the Sham group (P<0.05); these findings were compatible with the activation of the p38 MAPK/NF-κB pathway. The group treated with HF-SCS exhibited significantly reduced phosphorylation ratios compared with the SCI group (P<0.05) (Fig. 6B, G and H). In parallel, the expression levels of the proinflammatory cytokines TNF-α and IL-1β were significantly upregulated in the SCI group, which decreased again in the SCI + SCS group (P<0.05; Fig. 6A, E and F). These results collectively suggest that HF-SCS treatment may be associated with reduced CXCL10/CXCR3 expression, attenuated activation of the ROS/p38 MAPK/NF-κB pathway and decreased proinflammatory cytokine levels during the acute phase of SCI.

### HF-SCS promotes neuronal preservation and axonal remyelination at the chronic phase

**Figure 7:**
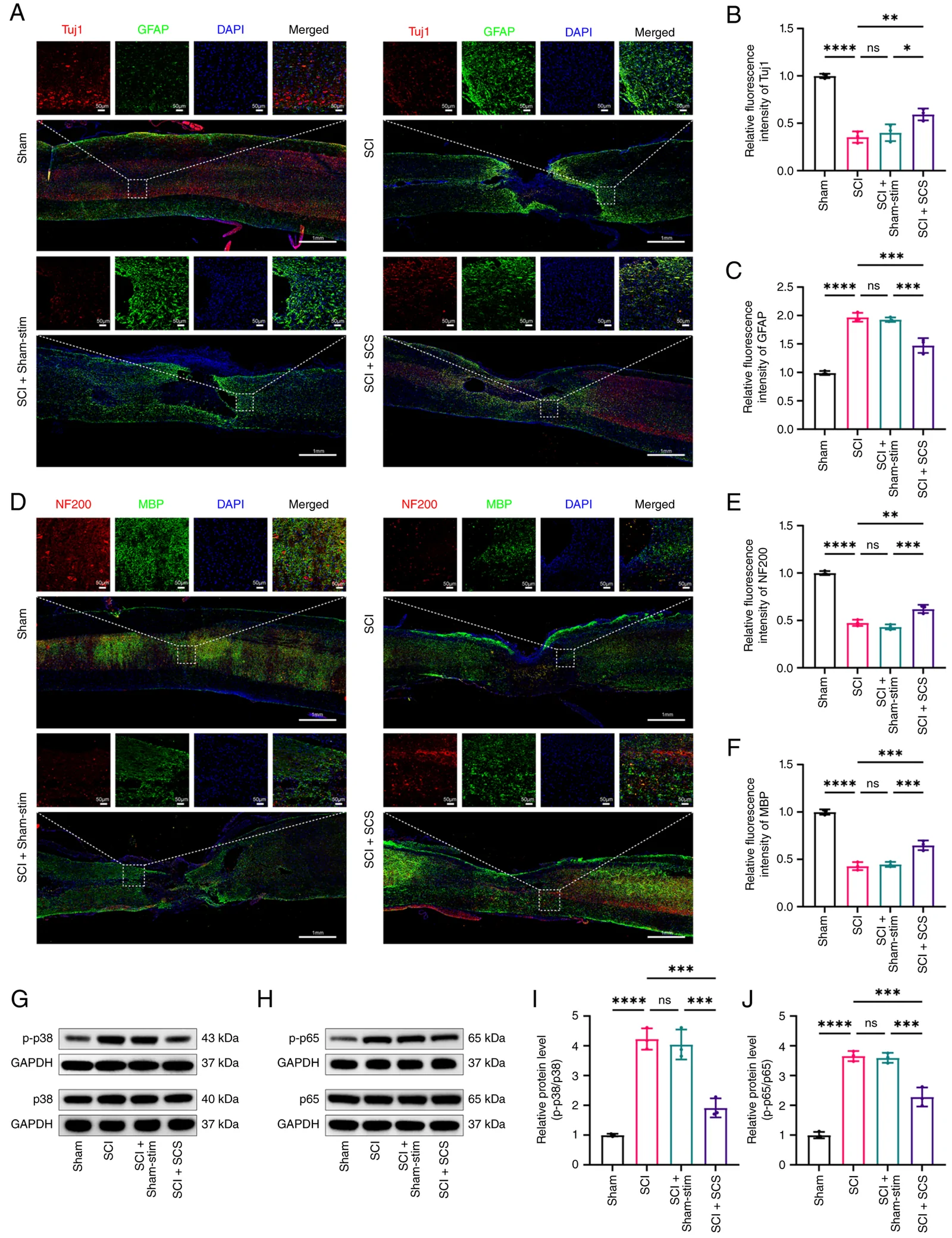
HF-SCS preserves neurons, promotes remyelination and suppresses persistent neuroinflammation at 35 dpi. (A) Representative images of immunofluorescence staining of the lesion epicenter showing Tuj1^+^ neurons (red) and GFAP^+^ astrocytes (green) at low (scale bar, 1 mm) and high (scale bar, 50 *μ*m) magnification. Quantification of (B) neuronal (Tuj1) and (C) astrocytic (GFAP) fluorescence intensity relative to that in the Sham group. (D) Representative images of immunofluorescence staining showing NF200 (red) and MBP (green) expression at low (scale bar, 1 mm) and high (scale bar, 50 *μ*m) magnification. Quantification of (E) axonal (NF200) and (F) myelin (MBP) fluorescence intensity relative to that in the Sham group. Representative western blots showing phosphorylated and total (G) p38 MAPK and (H) NF-κB p65 levels. Quantitative analysis of the (I) p-p38/p38 and (J) p-p65/p65 ratios. The data are presented as the mean ± SD (n=3). ^*^P<0.05, ^**^P<0.01, ^***^P<0.001, ^****^P<0.0001; one-way analysis of variance followed by Tukey's post hoc test. ns, not significant; DAPI, 4',6-diamidino-2-phenylindole; GFAP, glial fibrillary acidic protein; NF200, neurofilament-200; SCI, spinal cord injury; Tuj1, β-III tubulin; dpi, days post-injury; MBP, myelin basic protein; SCI, spinal cord injury; SCS, spinal cord stimulation; p-, phosphorylated.

Glial scarring in the region of SCI severely impedes directed axonal growth and synaptic reorganization through the formation of a dense physical barrier and the secretion of various inhibitory molecules (46). The reparative effects of HF-SCS on SCI were further characterized by examining nerve regeneration and glial scar formation at the lesion site using immunofluorescence staining at 35 dpi. Neurons and astrocytes were identified by Tuj1 and GFAP immunolabeling, respectively. Notably, the SCI + SCS group exhibited a marked aggregation of Tuj1^+^ neurons within the injury core, whereas a sparse neuronal distribution persisted in both the SCI and SCI + Sham-stim groups (Fig. 7A). The quantitative analysis confirmed that the Tuj1 fluorescence intensity was significantly higher in the SCI + SCS group than in the SCI and SCI + Sham-stim control groups (P<0.05; Fig. 7B). By contrast, GFAP^+^ astrocytes showed a notably higher fluorescence intensity in the SCI and SCI + Sham-stim groups, accompanied by prominent glial scar formation surrounding the lesion (Fig. 7A and C). Furthermore, double immunostaining for NF200^+^ and MBP^+^ revealed severe demyelination and axonal degeneration in SCI animals, pathological features that were notably ameliorated by HF-SCS intervention (Fig. 7D-F).

Western blot analyses of protein lysates from the spinal cord lesion site revealed sustained anti-inflammatory effects of HF-SCS at 35 dpi. Compared with both the SCI and SCI + Sham-stim groups, the SCS group had significantly lower p38 MAPK (p-p38/p38) and NF-κB p65 (p-p65/p65) phosphorylation ratios (P<0.05; Fig. 7G-J), indicating prolonged pathway inhibition at the injury epicenter. The persistent elevation of these phosphorylation markers in the SCI and SCI + Sham-stim groups suggested ongoing neuroinflammatory activity in the chronic phase. These findings demonstrate that HF-SCS maintained long-term suppression of p38 MAPK/NF-κB signaling at the lesion site, providing a sustained molecular basis for the neuroprotection and functional recovery observed at this chronic time point.

### HF-SCS alleviates NP by suppressing CXCR3 and p38 MAPK signaling in the DRG and SDH to attenuate central sensitization

**Figure 8:**
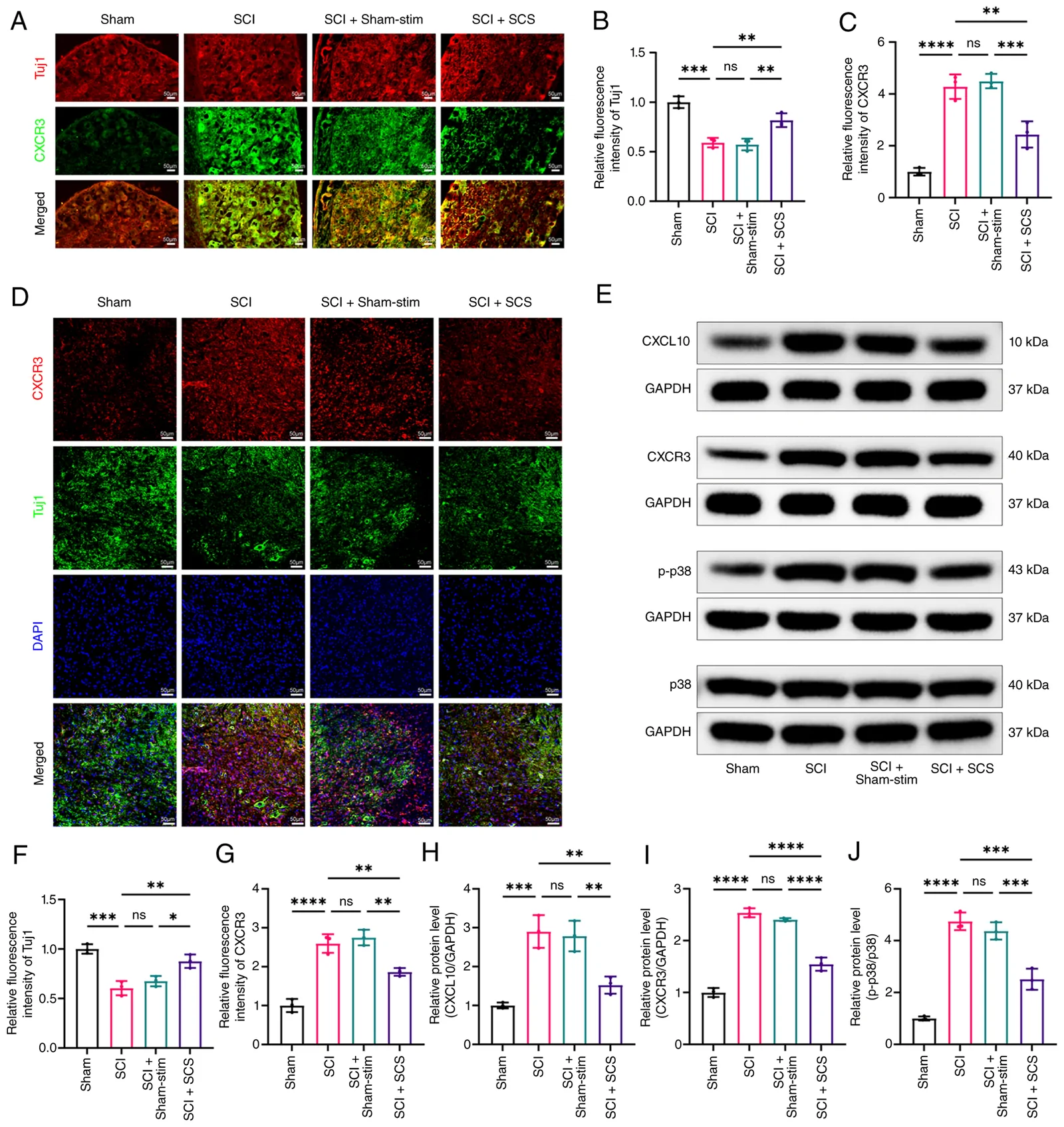
HF-SCS suppresses the activity of the CXCL10/CXCR3 axis and neuronal injury in the DRG and SDH. (A) Representative images of immunofluorescence staining of the DRG for Tuj1 (red, a neuronal marker) and CXCR3 (green) (scale bar, 50 *μ*m). Quantification of the fluorescence intensity of (B) Tuj1 and (C) CXCR3 in the DRG. (D) Representative images of immunofluorescence staining of the spinal dorsal horn for CXCR3 (red) and Tuj1 (green) (scale bar, 50 *μ*m). (E) Representative western blots showing CXCL10, CXCR3, total p38 and p-p38 levels in the DRG. Quantification of the fluorescence intensity of (F) CXCR3 and (G) Tuj1 in the SDH. Quantitative analysis of the levels of the following proteins in the DRG normalized to the levels of GAPDH: (H) CXCL10, (I) CXCR3 and (J) the p-p38/p38 ratio. The data are presented as the mean ± SD (n=3). ^*^P<0.05, ^**^P<0.01, ^***^P<0.001, ^****^P<0.0001; one-way analysis of variance followed by Tukey's post hoc test. ns, not significant; DAPI, 4',6-diamidino-2-phenylindole; dpi, days post-injury; HF-SCS, high-frequency spinal cord stimulation; DRG, dorsal root ganglion; SDH, spinal dorsal horn; SCI, spinal cord injury; Tuj1, β-III tubulin; p-, phosphorylated; CXCL10, C-X-C motif chemokine ligand 10; CXCR3, C-X-C motif chemokine receptor 3.

The activity status of sensory neurons in both the peripheral (DRG) and central (SDH) components of the nociceptive pathway were examined at 35 dpi to further elucidate the underlying mechanisms by which HF-SCS alleviates post-SCI NP. In the DRG, SCI induced marked neuronal loss (decreased Tuj1^+^ neurons) concomitant with CXCR3 upregulation; notably, HF-SCS effectively reversed these pathological alterations (P<0.05; Fig. 8A-C). Western blot analysis further confirmed that HF-SCS downregulated CXCL10 expression, CXCR3 expression and p38 MAPK phosphorylation in DRG tissue (P<0.05; Fig. 8E and H-J), indicating the suppression of the chemokine signaling cascade and peripheral sensitization at the primary afferent level. At the spinal level, the HF-SCS similarly attenuated CXCR3 immunoreactivity in the SDH while preserving the neuronal density (Fig. 8D, F and G). These convergent findings indicate that HF-SCS modulated CXCL10/CXCR3 and p38 MAPK signaling at multiple anatomical levels of the nociceptive pathway.

In pain research, the transcription factor c-Fos, an immediate early gene, has been widely used as a marker of neuronal activation in the SDH (47). At 35 dpi, c-Fos immunoreactivity was assessed as an indicator of neuronal activation. Compared with the Sham group, the number of c-Fos^+^ cells was significantly increased in the SCI group (P<0.05), indicating persistent central sensitization. HF-SCS treatment markedly reduced c-Fos expression (P<0.05), whereas sham stimulation did not exert a significant effect (P>0.05) (Fig. S3). These findings suggest that HF-SCS effectively suppressed aberrant neuronal activation in the SDH.

## Discussion

**Figure 9:**
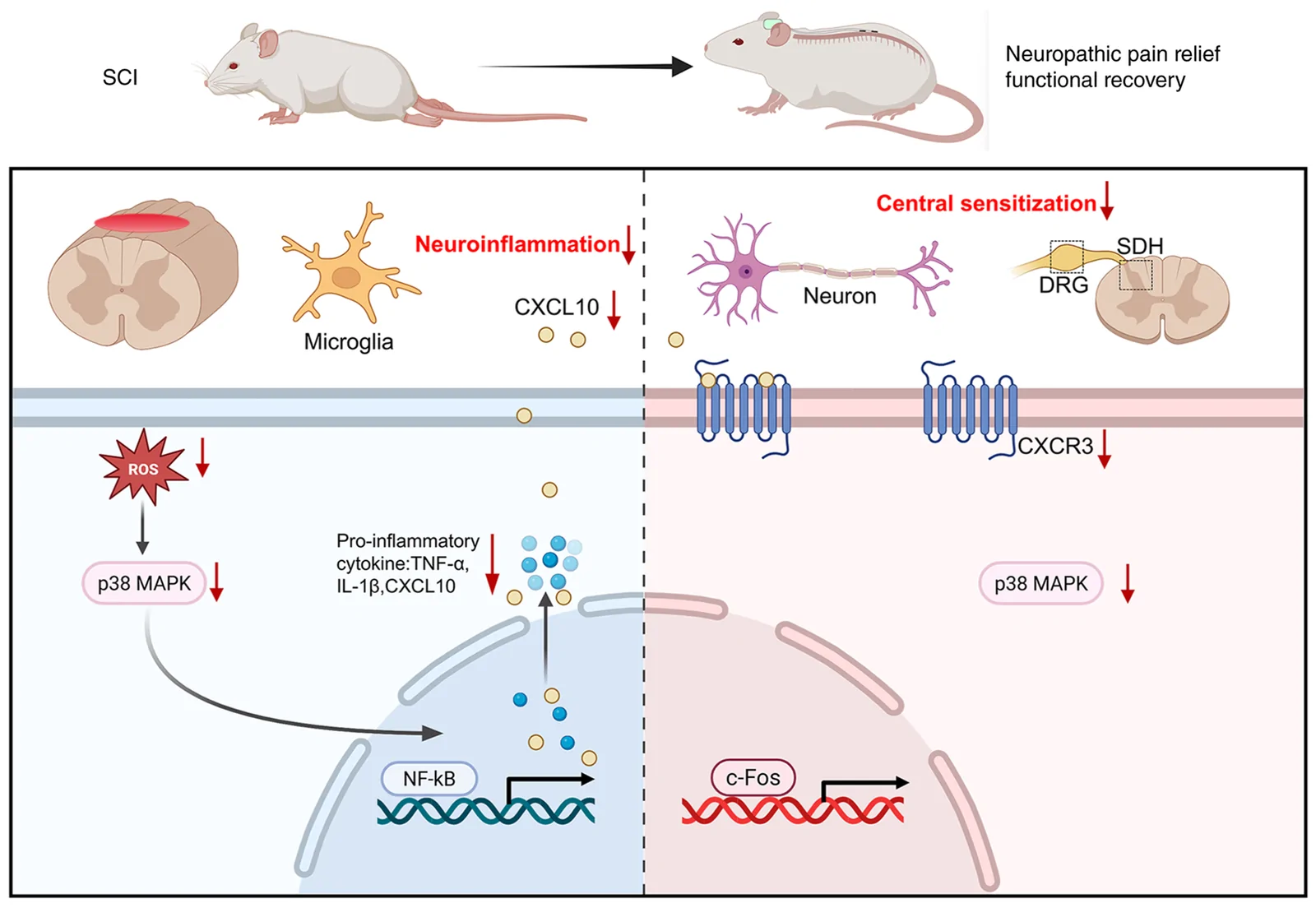
Proposed mechanism of high-frequency spinal cord stimulation in the treatment of SCI rats. SCI, spinal cord injury; ROS, reactive oxygen species; DRG, dorsal root ganglia; SDH, spinal dorsal horn; CXCL10, C-X-C motif chemokine ligand 10; CXCR3, C-X-C motif chemokine receptor 3. Created in BioRender. Liu, T. (2026) https://BioRender.com/a4itpn6.

The present study demonstrated that HF-SCS not only effectively alleviated post-SCI NP in rats but also promoted motor and neurological functional recovery. The underlying mechanisms mainly included two aspects. First, HF-SCS suppressed acute-phase neuroinflammation by inhibiting microglial activation and proinflammatory cytokine release via the ROS/p38 MAPK/NF-κB and CXCL10/CXCR3 pathways, thereby improving the neural microenvironment. Second, HF-SCS attenuated central sensitization in the SDH through CXCL10/CXCR3 inhibition, effectively ameliorating NP. These sequential actions not only relieved NP but also facilitated motor and neurological recovery after SCI. A schematic diagram illustrating this integrated mechanism is presented in Fig. 9.

In the present study, HF-SCS markedly reduced c-Fos expression, microglial activation markers and inflammatory protein levels, although complete normalization to sham levels was not achieved. This outcome was consistent with the chronic and irreversible nature of SCI, where established neuroinflammatory changes are difficult to fully reverse. Notably, the partial restoration of spinal microenvironment homeostasis significantly alleviated NP and improved motor function, supporting the translational potential of this intervention.

Secondary neuroinflammation is a critical driver of tissue damage and NP after SCI (7). Previous studies have demonstrated that upon SCI, the rapid accumulation of ROS activates the p38 MAPK/NF-κB signaling axis, triggering the release of proinflammatory cytokines (such as TNF-α, IL-1β and IL-6) and exacerbating neuronal and glial damage (8,48). Microglial activation contributes directly to neuroinflammation and central sensitization, thereby promoting NP and motor dysfunction (49,50). While the pathological role of the ROS/p38 MAPK/NF-κB pathway in SCI has been well established (8,13), its modulation by SCS (particularly HF-SCS) has remained poorly characterized. The findings of the present study indicate that HF-SCS suppressed microglial activation, at least in part, by inhibiting the ROS/p38 MAPK/NF-κB signaling cascade in these cells. This result was further supported by the reduced phosphorylation of p65 at Ser536 (p-p65), a key subunit of the NF-κB transcription complex whose phosphorylation at Ser536 is critical for its transcriptional activity, suggesting that HF-SCS suppressed NF-κB activation at the molecular level (51). This finding was consistent with prior observations that electrical neuromodulation can modulate neuroimmune responses (20,52) and specifically implicates the ROS/p38 MAPK/NF-κB axis as a therapeutic target in post-SCI neuroinflammation. Notably, while conventional low-frequency SCS has been reported to increase M1-like microglial activity (22), the high-frequency paradigm employed in the present study appeared to exert the opposite effect, suggesting that the stimulation frequency may critically affect glial phenotypes.

In the present study, the functional significance of NF-κB inhibition by HF-SCS was further supported by the downregulation of canonical NF-κB target genes, including TNF-α and IL-1β, observed at 7 dpi. These proinflammatory cytokines are established mediators of secondary injury propagation, and their suppression is expected to foster a microenvironment conducive to neural repair (53). Taken together, the findings of the present study extend prior work on the electrical neuromodulation of neuroimmune responses by pinpointing the ROS/p38 MAPK/NF-κB axis as a specific molecular substrate for HF-SCS-mediated neuroprotection in the injured spinal cord.

Central sensitization, which is characterized by hyperexcitability of SDH neurons and facilitated nociceptive transmission, constitutes a key mechanism underlying chronic NP after SCI (54,55). The chemokine CXCL10 and its receptor CXCR3 have been increasingly recognized as key mediators of NP in peripheral nerve injury models, where they increase neuronal excitability and activate downstream p38/ERK signaling in nociceptive pathways (15). However, existing studies on the CXCL10/CXCR3 axis have focused predominantly on NP induced by peripheral nerve injury (18,56), while its involvement in central NP following direct SCI remains to be fully elucidated. The present study found that CXCR3 was expressed on DRG neurons (Tuj1^+^) and that SCI induced the significant upregulation of CXCL10/CXCR3 expression together with increased p38 phosphorylation in the DRG. In the SDH, SCI increased CXCR3^+^/Tuj1^+^ and c-Fos^+^ neurons, reflecting increased neuronal excitability. Treatment with HF-SCS markedly attenuated all these molecular and cellular changes in both the DRG and SDH, concomitant with reduced mechanical and thermal hypersensitivity. By reducing the DRG-derived nociceptive drive and suppressing SDH hyperexcitability, HF-SCS likely disrupts the positive feedback loop that maintains central sensitization. These data identify a previously underappreciated analgesic mechanism of HF-SCS and position the CXCL10/CXCR3 pathway as a potential therapeutic target in post-SCI NP.

The ability of epidural SCS to restore locomotor function after SCI has been extensively investigated. Previous studies, primarily those employing low-frequency (tonic or burst) paradigms, have demonstrated that SCS can facilitate motor recovery by recruiting dorsal root afferents, activating spinal locomotor networks, and enhancing residual descending drive (57). However, these investigations have largely focused on immediate or subacute electrophysiological responses, with limited attention given to chronic-phase histological remodeling (58). A notable finding of the present study is that the anti-inflammatory and anti-sensitization effects of HF-SCS were translated into sustained structural neuroprotection during the chronic phase (35 dpi). Specifically, significantly reduced astrocyte activation (decreased GFAP expression), increased neuronal survival (increased Tuj1-positive neurons), preserved axonal integrity (increased NF-200 immunoreactivity) and the induction of remyelination (increased MBP expression) were observed in the perilesional region of HF-SCS-treated rats. These histological improvements provide a structural substrate for the observed long-term gains in motor and neurological function. A prior study on the effects of SCS on SCI has primarily documented functional improvements without corresponding chronic-phase histological evidence (26). The results of the present study suggest that by dampening early neuroinflammation, HF-SCS mitigates the glial scar formation and demyelination that typically impede axonal regeneration and circuit reorganization in chronic SCI.

In the present study, transcriptomic analyses (KEGG and GSEA) revealed that HF-SCS significantly reversed the injury-induced upregulation of multiple pathways associated with inflammation. These changes were consistent with the histological and behavioral findings, indicating that HF-SCS reshaped the spinal microenvironment from a proinflammatory state toward a repair-permissive state. Thus, the anti-inflammatory effect of HF-SCS was not transient but enduring, remaining active at 35 dpi and contributing to long-term functional recovery. These findings expand the concept of the therapeutic window for SCS, suggesting that early intervention may exert a lasting effect on the pathological microenvironment by shifting the inflammatory response from a destructive state toward a state that promotes repair and neuroplasticity.

The present findings demonstrated the safety and therapeutic efficacy of HF-SCS over a 35-day treatment period in a rat model of SCI. Terminal histopathology at 35 dpi revealed no thermal necrosis, peri-electrode fibrosis or aberrant glial reactivity, supporting the biocompatibility of this protocol. Compared with both the SCI and SCI + Sham-stim groups, the SCI + SCS group showed significant improvements in locomotor function, NP relief and the levels of molecular biomarkers, underscoring the neurorestorative potential of electrical neuromodulation. Moreover, the equivalent ROS levels, microglial activation and behavioral outcomes between the SCI and SCI + Sham-stim groups indicate that electrode implantation alone did not induce secondary pathology, excluding its confounding influence on the therapeutic effects. Compared with pharmacological immunosuppressants or opioids, which carry substantial risks of tolerance and dependency (5), HF-SCS represents a localized neuromodulatory approach with a markedly superior adverse effect profile. The present study has several limitations that should be noted. First, although the mechanistic analyses identified the ROS/p38 MAPK/NF-κB and CXCL10/CXCR3 pathways as key mediators of the effects of HF-SCS, gene knockout models or pharmacological blockade were not employed to establish definitive causality. Additionally, while the RNA-seq data supported microenvironmental remodeling, cell type-specific transcriptional changes require further validation using single-cell sequencing. Second, the present study was conducted exclusively in female rats; given the well-documented sex-dependent dimorphism in neuroimmune responses to NP (59,60), whether these findings extend to male animals remains unknown. Future studies are therefore warranted to validate whether the anti-inflammatory and analgesic effects of HF-SCS observed are reproducible in male rats. Third, the 35-day observation period aligned with established SCI recovery timelines, where spontaneous locomotor recovery typically plateaus by 4 weeks post-injury (61). However, this duration is insufficient to assess long-term stimulation-related complications, including tolerance development and a decrease in efficacy, which may manifest only after extended treatment periods. Future studies with longer observation windows are warranted to validate the chronic safety and sustained therapeutic effects of HF-SCS.

In conclusion, the present study provides compelling evidence that HF-SCS exerts neuroprotective effects through the coordinated modulation of the p38 MAPK/NF-κB and CXCL10/CXCR3 pathways, thereby suppressing neuroinflammation and attenuating central sensitization following SCI. These findings underscore the therapeutic potential of an early neuromodulatory intervention for promoting functional recovery and NP relief. Moreover, the results advance the understanding of molecular cascades underlying secondary injury progression and support the clinical translation of HF-SCS as a viable strategy for SCI repair.

## Supplementary Data



## Data Availability

The RNA sequencing data generated in the present may be found in the NCBI Gene Expression Omnibus database under accession number GSE332999 or at the following URL: https://www.ncbi.nlm.nih.gov/geo/query/acc.cgi?acc=GSE332999. All other data generated in the present study may be requested from the corresponding author.
