# Supplementary material for: Early high-frequency spinal cord stimulation modulates the ROS/p38 MAPK/NF-κB and CXCL10/CXCR3 pathways to alleviate neuropathic pain and promote spinal cord injury repair
Source: Int J Mol Med. 2026 Aug 13;58(4):285. doi: 10.3892/ijmm.2026.5956 (PMC13502485; doi:10.3892/ijmm.2026.5956)

Figure S1. HF-SCS improves fine motor function. (A) Schematic of the automated home cage monitoring system. Proportion of time spent (B) rearing, (C) grooming and (D) jumping. The data are presented as the mean ± SD (n=6). $^{*}P<0.05$, $^{***}P<0.001$, $^{****}P<0.0001$; one-way analysis of variance followed by Tukey's post hoc test. ns, not significant; HF-SCS, high-frequency spinal cord stimulation; SCI, spinal cord injury.

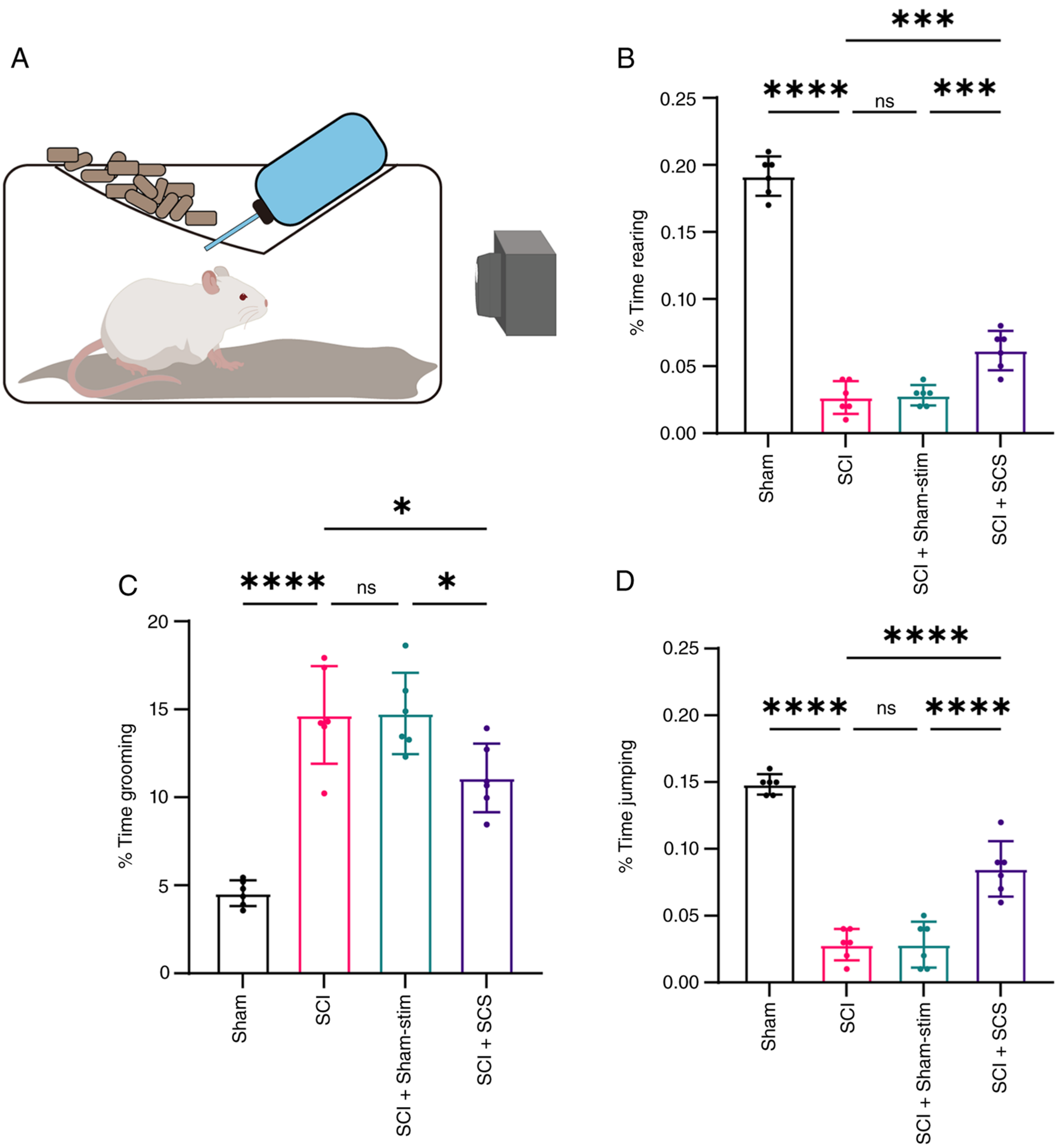

Figure S2. HF-SCS modulates gene expression and suppresses neuroinflammatory pathways. (A) Differences in gene transcription between the SCI + SCS group and the SCI group are shown in the heatmap. GSEA of upregulated pathways following SCI (SCI vs. Sham group): (B) chemokine signaling, (C) cytokine-cytokine receptor interaction and (D) NF-κB signaling. GSEA of pathways downregulated by HF-SCS treatment (SCI + SCS vs. SCI group): (E) NF-κB signaling and (F) cytokine-cytokine receptor interaction (n=3). GSEA, Gene Set Enrichment Analysis; HF-SCS, high-frequency spinal cord stimulation; NES, normalized enrichment score; SCI, spinal cord injury.

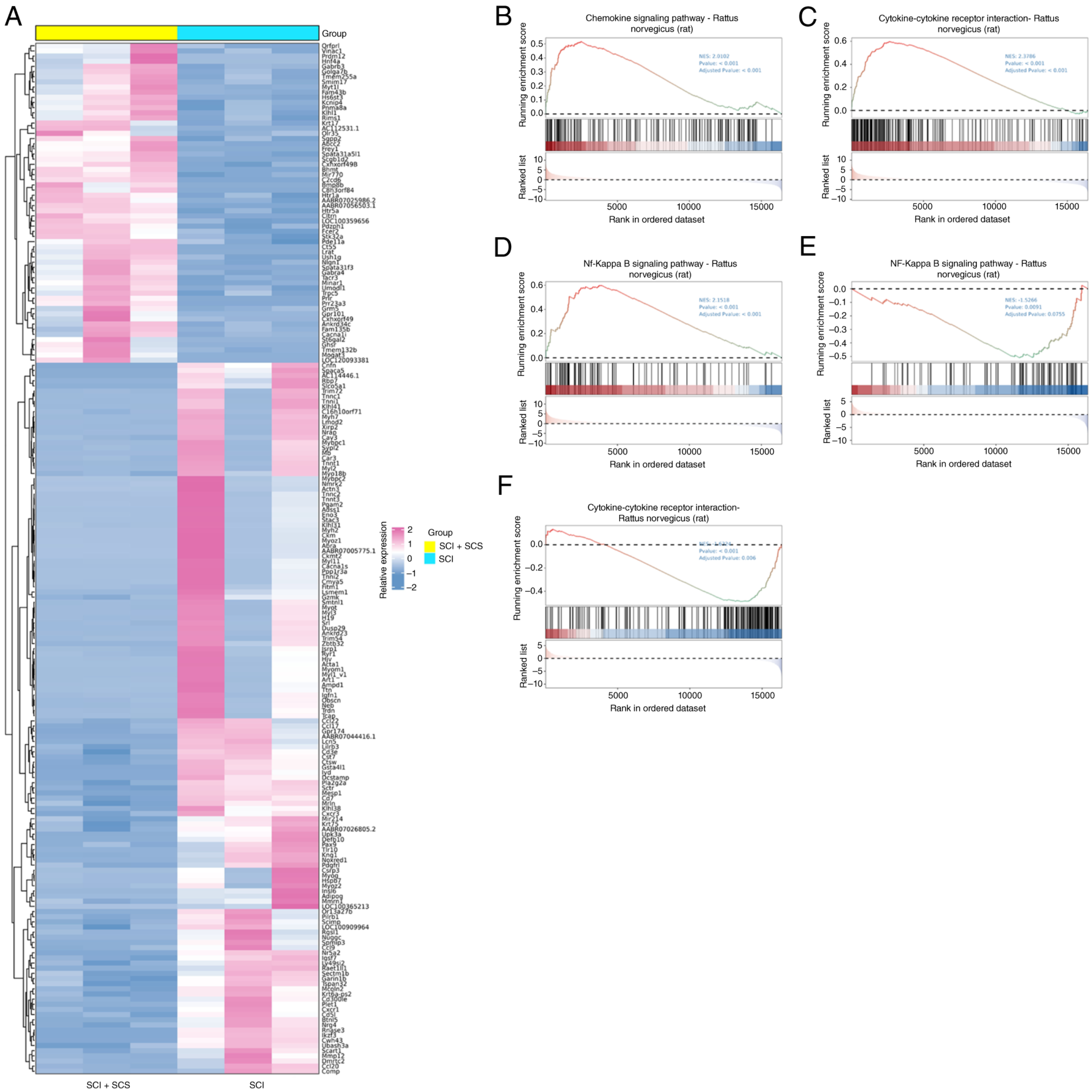

Figure S3. HF-SCS attenuates c-Fos activation in the SDH at 35 dpi. (A) Representative images of c-Fos immunofluorescence staining in the SDH at 35 dpi (scale bar, 50 μm). (B) Quantification of c-Fos$^+$ cells. The data are presented as the mean ± SD (n=3). **P<0.01, ****P<0.0001; one-way analysis of variance followed by Tukey's post hoc test. ns, not significant; dpi, days post-injury; HF-SCS, high-frequency spinal cord stimulation; SDH, spinal dorsal horn; SCI, spinal cord injury; DAPI, 4',6-diamidino-2-phenylindole.

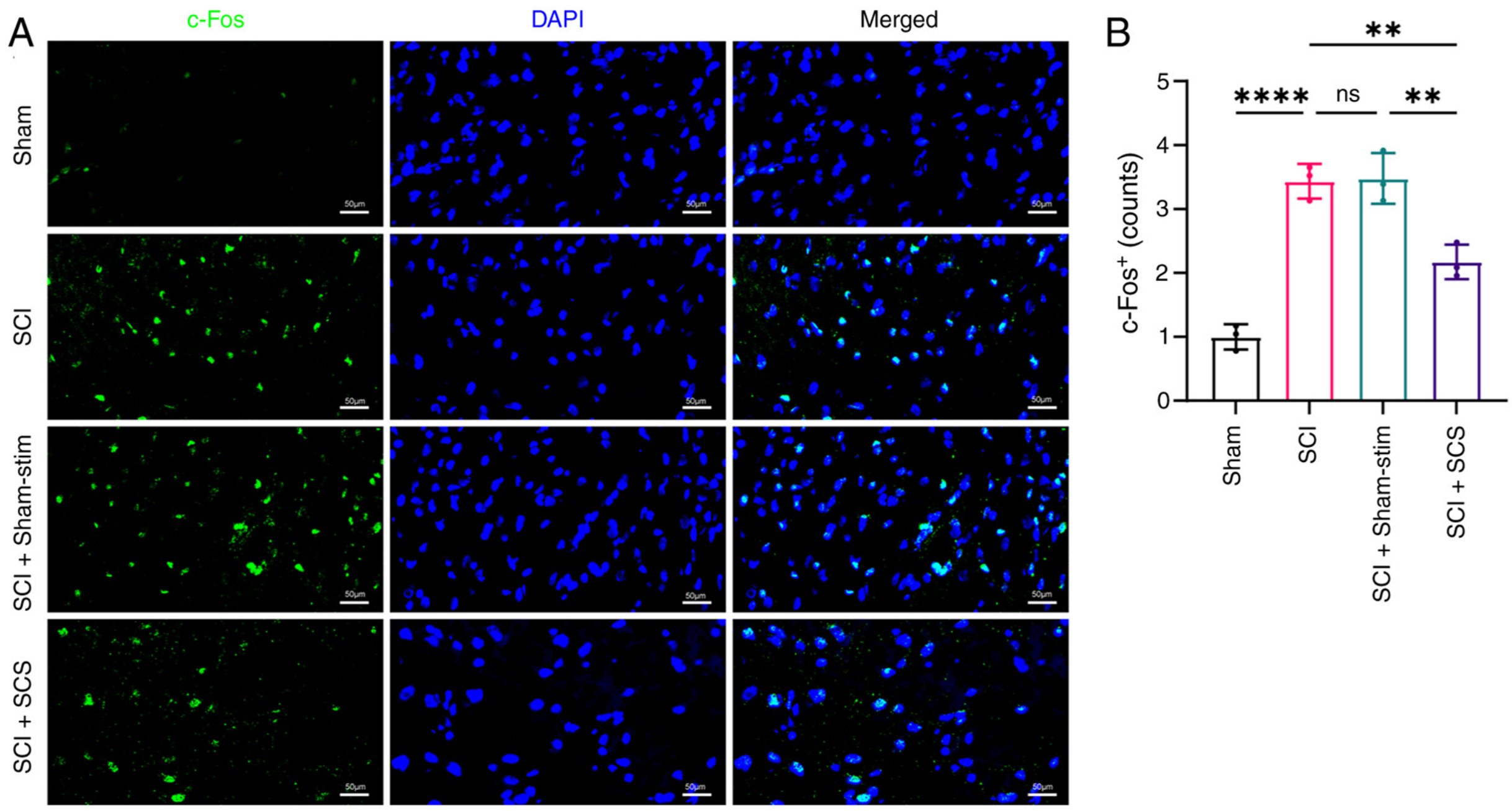

Supplement: Supplementary file 1 [file Supplementary_Data.pdf]
